# Sociodemographic and Obstetric Characteristics of Anaemic Pregnant Women Attending Antenatal Clinic in Bolgatanga Regional Hospital

**DOI:** 10.1155/2016/4687342

**Published:** 2016-05-03

**Authors:** Benjamin Ahenkorah, Kwabena Nsiah, Peter Baffoe

**Affiliations:** ^1^Haematology and Parasitology Units, Bolgatanga Regional Hospital, P.O. Box 26, Bolgatanga, Upper East Region, Ghana; ^2^Department of Biochemistry and Biotechnology, Kwame Nkrumah University of Science and Technology, Kumasi, Ghana; ^3^Obstetric Gynaecology Unit, Bolgatanga Regional Hospital, P.O. Box 26, Bolgatanga, Upper East Region, Ghana

## Abstract

The study determined the sociodemographic and obstetric characteristics of pregnant women which contribute to the risk of developing anaemia. A cross-sectional study was conducted among 400 pregnant women attending their first antenatal visit at the Bolgatanga Regional Hospital Antenatal Clinic. Anaemia was significantly associated (*p* < 0.05) with younger maternal age, parity, gravidity, trimester of pregnancy, and source of drinking water. Multivariate logistic regression identified the following factors with adjusted odds ratios (aOR) and 95% confidence intervals (CI): unemployment (aOR = 4.76 (CI: 2.26–11.33); *p* < 0.0001), rural dwelling (aOR = 3.10 (CI: 2.16–4.91); *p* = 0.0071), primigravida (aOR = 2.13 (CI: 1.34–3.18); *p* = 0.0201), nulliparity (aOR = 1.92 (CI: 1.23–2.86); *p* = 0.0231), first antenatal visit at second trimester (aOR = 1.71 (CI: 1.33–3.12); *p* = 0.0149) and first antenatal visit at third trimester (aOR = 2.73 (CI: 1.24–4.35); *p* = 0.0017), drinking from well and boreholes (aOR = 2.78 (CI: 2.27–5.21); *p* < 0.0001), and the presence of domestic livestock (aOR = 2.15 (CI: 1.33–3.68); *p* = 0.0019). This study has shown the various sociodemographic and obstetric factors which significantly contribute to anaemia in pregnancy.

## 1. Introduction

Anaemia in pregnancy (haemoglobin level <11 g/dL, as defined by World Health Organization [1] and haemoglobin level <10 g/dL as defined by the Ministry of Health of Ghana [2]) is a major public health problem, especially in developing countries. Recent statistics indicate that anaemia affects 41.8% of pregnant women globally, with the highest prevalence in Africa [3]. Fifty-seven percent of pregnant women in Africa are anaemic, which corresponds to ~17 million affected women, with severe consequence on health and socioeconomic development [1, 4–6].

Anaemia in pregnancy is associated with negative consequence for both the woman and neonate. Foetal anaemia, low birth weight (LBW), preterm birth, low APGAR score, intrauterine growth restriction, and perinatal mortality have been associated with anaemia [6–12]. In the women themselves it may cause low physical activity and increased risk of maternal morbidity and mortality, especially in those with severe anaemia [1, 6, 7].

The cause of anaemia in pregnancy is multifactorial. Low caloric intake, leading to deficiencies in iron, folate, vitamin B_12_, and vitamin A, and intestinal parasitic infections, malaria, haemoglobinopathies, and HIV have all been shown to be the main causes of anaemia, among pregnant African women [6, 8, 13–16].

Nulliparity and grand multiparity, low socioeconomic status, young age, illiteracy, low birth spacing, and cultural factors, such as taboos prohibiting pregnant women from consuming meat or egg-based foods also predispose pregnant women in developing countries to anaemia [17–19]. These predisposing factors are not universal in their significance. Their significance varies from one antenatal population to another. Within the same population, not all of them might even be significant.

Anaemia remains a major public health problem in Ghana and more effort is needed to combat it. In 2003, according to the GDHS survey, the prevalence of anaemia among children aged 6–59 months was 76% and that of severe anaemia was 6%. Prevalence of anaemia was significantly higher in rural areas (80%) than in urban areas (68%). By region, there were substantial disparities, ranging from a prevalence of 61% in Greater Accra Region to 83% in Northern Region [20]. Overall, the prevalence of anaemia was higher in rural areas than in urban areas and disparities across regions were marked, ranging from 34% in Brong Ahafo Region to 51% in Upper East Region [20].

This cross-sectional study was designed to determine whether significant changes in sociodemographic and obstetric characteristics contribute to low haemoglobin levels of anaemic pregnant women attending Antenatal Clinic at the Bolgatanga Regional Hospital.

## 2. Materials and Methods

### 2.1. Study Design and Setting

This cross-sectional study was conducted at the Obstetrics and Gynaecology Unit of the Bolgatanga Regional Hospital, from May 2013 to May 2014. Bolgatanga is the capital town of both the Bolgatanga Municipal Assembly and the Upper East Region of Ghana. It is situated at the centre of the region and to the north-eastern part of Ghana. It has a total land area of 729 sq km. It is bounded to the north by Bongo District, to the south and east by Talensi-Nabdam District, and to the west by the Kassena-Nankana District (http://www.bolga.ghanadistricts.gov.gh).

Bolgatanga Municipal Assembly has an estimated population of 38,083 women in fertility age group (WIFA) and 6,347 expected pregnancies (according to 2012 projections). The municipality has one regional hospital, 3 health centres, 6 functional community health integrated centres, and 10 clinics (two are privately owned).

The Bolgatanga Regional Hospital (RHB) was established in 1946 to serve the minority white population of the then Gold Coast. It is a regional referral hospital and training centre for housemen and junior doctors, nurses, midwives, laboratory technologists, biomedical scientists, dentists, radiographers, and pharmacists. It provides both primary healthcare and some specialist referral services and it has a catchment area population of about 1,004,243 (according to 2011 annual projections). The antenatal coverage for the Upper East Region for the year 2011 was 84.9% (5,301), and that for the Bolgatanga Regional Hospital was 19% (1233) of the 6,347 municipality's expected pregnancies. Thus, hospital-based samples give a fair representation of the total antenatal population in the region.

### 2.2. Study Population

The study population was 200 anaemic pregnant women of haemoglobin concentration <10 g/dL, as test, and 200 nonanaemic pregnant women of haemoglobin concentration >10 g/dL, as control. The study included all pregnant women attending Antenatal Clinic at the Bolgatanga Regional Hospital. Pregnant women in need of emergency care or having an at-risk pregnancy such as gestational diabetes, preeclampsia, and eclampsia, antenatal pregnant women reporting for repeat visits during the study period, were excluded from the study. Pregnant women attending their first antenatal visit at the RHB-ANC were approached and the rationale of the study was explained to them.

### 2.3. Informed Consent and Subject Recruitment

Pregnant women attending their first antenatal visit at the RHB-ANC were approached and informed consent was then sought from subjects. Using a structured questionnaire, their sociodemographic data were obtained. In the process of seeking informed consent, the aims and objectives of the study, as well as the benefits of the proposed study, were explained to the participants.

### 2.4. Ethical Review

The ethical approval of the research protocol was granted by two review boards. Firstly, the Committee on Human Research, Publication, and Ethics of Kwame Nkrumah University of Science and Technology and Komfo Anokye Teaching Hospital (CHRPE-KNUST/KATH) reviewed and approved it and secondly it was reviewed and approved by the Institutional Review Board of the Navrongo Health Research Centre (IRB-NHRC).

### 2.5. Specimen Collection and Transfer

About 2 mL of participants' venous blood was drawn into a BD vacutainer, containing EDTA for the determination of haemoglobin concentration. The collected samples were transferred in a cold box to the Haematology laboratory of RHB for haemoglobin determination. About 2 g of early morning stool and 10 mL midstream urine samples were also collected into sterile containers. The urine was used for the determination of* Schistosoma haematobium* and the stool for the determination of enteric parasites.

### 2.6. Haemoglobin Determination

Haemoglobin concentration was determined using the Sysmex KX-21N Automated Haematology Analyzer (Sysmex Corporation Kobe, Japan), Whole Blood Mode, and so forth.

### 2.7. Stool and Urine Analysis

Stool and urine samples were analysed to detect enteric parasites in stool and* Schistosoma haematobium* in urine. The formol-ether concentration method was used in the preparation of stool samples for microscopy and detection of helminthes. The urine sedimentation technique was used to detect the presence of* S. haematobium* ova.

### 2.8. Malaria Parasite Screening

The examination of the blood film for malaria parasites was done by two certified microscopists independently. If there was nonconcurrence in presence of parasite and level of parasite density between the primary readers, the sample was referred to a third expert microscopist whose determination of parasitaemia was considered final. The thick smear was used to examine each slide so as to detect very mild infection with scanty number of parasites. The slides were examined using the Primo Star (Carl Zeiss MicroImaging GmbH, Germany) microscope with the ×100 objective lens. The parasitaemia for positive slides was determined using the plus (+) system of quantification. The results were categorised as follows: 1–9 parasites per 100 microscopic fields (+); 10–99 parasites per 100 microscopic fields (++); 1–9 parasites per microscopic field (+++); more than 10 parasites per microscopic field (++++). Slides were declared negative when 100 high power fields were scanned without any parasite being seen.

### 2.9. Statistical Analysis

Data were entered into Microsoft Excel worksheet. Results were presented as mean ± standard deviation (SD) and frequency (percentage), where necessary. The Fischer's exact test or Chi-square (*χ* ^2^) was used to assess the statistical significance of categorical variables. A *p* value less than 0.05 was considered statistically significant. Multivariate logistic regression was used to predict associated risk factors. Analysis was performed using GraphPad Prism 5 Project software (GraphPad software, San Diego, California, USA, http://www.graphpad.com/).

## 3. Results

### 3.1. Sociodemographic and Obstetric Characteristics of Study Population

According to Table 1, the mean ages of the anaemic and nonanaemic pregnant women were similar, thus 27.43 and 27.52 years, respectively. The proportion of younger women dominated (76.5%), compared to only 23.5% older women who were anaemic. A higher number of the nonanaemic subjects were from the urban setting, while more anaemic women were from rural settlement (60.0%).

For both the anaemic and nonanaemic subjects, the majority had basic education, followed by those who were illiterate. Participants with higher education were more likely to be nonanaemic than to be anaemic. The test population and control were both predominantly self-employed. Whereas there was a significantly higher proportion of unemployed subjects in the anaemic women (27.0%), there was a lower proportion (5.5%) of civil servants who had anaemia.

A majority of the nonanaemic women were multigravida, while a significantly higher percentage of primigravid women (39.5%) were anaemic. Even though significantly higher percentage of both test and control were multiparous, the nonanaemic women had the higher multiparity. On the other hand, the anaemic women were significantly primiparous. In the case of the nonanaemic women, the highest percentage reported to the ANC in the first trimester of their pregnancy, but in the anaemic women, the majority reported in the second trimester. Between the test and control populations, there was no difference in percentages recruited in the third trimester of pregnancy. A higher proportion of the anaemic pregnant women (70.5%) drank from well and borehole and other sources, compared to 41.0% of the nonanaemic control (*p* < 0.0001), but the main source of water supply for the nonanaemic control was pipe-borne.

Crude odds ratios from the logistic regression analysis showed the following: maternal age <30 years (aOR = 1.677; 95% CI (1.081–2.601); *p* = 0.0269), unemployment (aOR = 5.058; 95% CI (2.258–11.33); *p* < 0.0001), primigravida (aOR = 2.067; 95% CI (1.344–3.181); *p* = 0.0020), nulliparity (aOR = 1.879; 95% CI (1.234–2.861); *p* = 0.0043), first antenatal visit at second trimester (aOR = 2.036; 95% CI (1.327–3.123); *p* = 0.0013) and first antenatal visit at third trimester of pregnancy (aOR = 2.319; 95% CI (1.236–4.349); *p* = 0.0012), usage of both well and borehole (aOR = 3.44; 95% CI (2.27–5.21); *p* < 0.0001), and rural settlement (aOR = 3.26; 95% CI (2.162–4.92)) were significantly associated with anaemia.

### 3.2. Nutrition, BMI, and Source of Drinking Water of Study Participants

From Table 2, whether in the anaemic or nonanaemic women, most of the participants took meals three times daily. However, a higher proportion of anaemic pregnant women ate from both home and street, compared to nonanaemic controls (59.5% versus 41.5%; *p* < 0.0001). Those who took meals from their homes predominated in the nonanaemic group.

The presence of domestic livestock was reported in the test and control subjects, but it was more common in those who were anaemic. Body mass index was similar in the two groups of pregnant women (*p* > 0.05). Crude odds ratios in the logistic regression analysis indicated those who ate from both home and street had higher odds (aOR = 1.98, 95% CI (1.31 to 2.97); *p* = 0.0011), likewise presence of domestic livestock (aOR = 2.15, 95% CI (1.33 to 3.68); *p* = 0.0027) (Table 2).

### 3.3. Association between Anaemia and Parasitaemia

Higher proportion of women with anaemia had malaria parasitaemia and intestinal parasitic infections. There was no statistically significant association between anaemia and malarial parasitaemia (*p* = 0.794) as well as intestinal flagellates (*p* = 0.137). All schistosomiasis infections were found in anaemic participants.

Table 4 shows multivariate logistic regression analysis. After adjusting for advanced maternal age, the independent risk factors for anaemia gave the following adjusted odds ratios and their 95% confidence intervals: unemployment (aOR = 4.76 (CI: 2.26–11.33); *p* < 0.0001), rural settlement (aOR = 3.10 (CI: 2.16–4.91); *p* = 0.0071), primigravida (aOR = 2.13 (CI: 1.34–3.18); *p* = 0.0201), nulliparity (aOR = 1.92 (CI: 1.23–2.86); *p* = 0.0231), first antenatal visit in second trimester (aOR = 1.71 (CI: 1.33–3.12); *p* = 0.0149) and visit in third trimester (aOR = 2.73 (CI: 1.24–4.35); *p* = 0.0017), drinking from well and boreholes (aOR = 2.78 (CI: 2.27–5.21); *p* < 0.0001), and the presence of domestic livestock (aOR = 2.15 (CI: 1.33–3.68); *p* = 0.0019).

## 4. Discussion

This study was aimed at evaluating the sociodemographic and obstetric characteristics of anaemic pregnant women attending Antenatal Clinic at the Bolgatanga Regional Hospital. The results of this study indicated that anaemia was significantly associated with age, parity, gravidity, presence of domestic livestock, and trimester of pregnancy when the first antenatal visit was made. Independent risk factors of anaemia were unemployment, rural dwelling, primigravida, nulliparity, drinking from well and borehole, and presence of domestic livestock.

From Table 1, there was a significant association between maternal age and anaemia, as a higher proportion of younger pregnant women (76.5%) were anaemic. The greater susceptibility of younger women to anaemia can be attributed to the fact that younger pregnant women belong to the physically active group, undergoing rapid growth and having increased nutritional requirements [21, 22]. Because of the increased iron requirements of pregnancy and growth, pregnant women, especially the younger ones and infants, are recognized as the groups most vulnerable to iron deficiency anaemia.

The observation that 33.5% of the anaemic women were illiterates and 50.0% had only basic education implies that such women probably did not have adequate knowledge of proper nutrition and balanced diet. Participants from the rural settlements were more anaemic (60.0%), compared to those from urban settlements (40.0%). This is consistent with the findings of Eweh [23] and Glover-Amengor et al. [2] who assessed the determinants of anaemia among Ghanaian pregnant women. They observed that anaemia was significantly higher among rural dwellers, compared to urban dwellers. According to our study, participants who lived in the rural settlements were 3.26 times more likely to be anaemic, compared to their counterparts from the urban settlements.

A number of factors may have contributed to anaemia among the rural settlers. Rural settlements, especially in the northern part of Ghana, depend solely on streams, wells, and dug-out dams, which are the major sources and breeding sites for mosquitoes and intestinal parasites. Thus living in urban areas significantly decreases the risk of anaemia in pregnancy. Most of the inhabitants of urban areas, approximately 68.5%, were nonanaemic pregnant women having higher educational qualification and well-paid jobs.

This study has also shown that participants with parasitic infections such as malaria and intestinal parasites (intestinal flagellate,* S. mansoni*, and* S. haematobium*) were more anaemic (Table 3), though the difference was not statistically significant. This is in contrast to a study by Agu et al. [24] who observed a significant association between parasitic infection and anaemia among pregnant women. Majority of women with anaemia were from the rural settlement and the presence of intestinal parasite and malarial infection could have contributed to the anaemia. Living in the rural areas had a significant association with anaemia (Table 1). This might be probably due to, although not significant, frequent exposure to malaria infections (Table 3). The smaller sample size (200 anaemic population) recruited for the study could have influenced the findings on malaria infection. A similar research conducted elsewhere had significant association between malaria infection and anaemia, partly due to the larger sample size used [25]. Malaria infections were predominant in the anaemic pregnant women because most rural settings in the Upper East Region have houses made of mud walls and thatch roof (data on housing not shown).

The association between housing and malaria has been described previously in Africa and elsewhere [26–28]. In Eritrea, walls made from mud increased an individual's risk for malaria parasitaemia, compared to individuals living in houses with walls made of other construction materials [29]. An earlier study in Eritrea also revealed an association between mud walls and malaria infection [30]. These types of housing construction provide microenvironments for mosquitoes and may ensure their chance of survival and feeding opportunities [31].

A significant association was also found between the prevalence of anaemia and gravidity in this study. According to Desalegn, there was a twofold decrease in the risk of anaemia as the number of pregnancies increases [32]. The findings of Desalegn concurs well with our study which observed that primigravida pregnant women had about 2 times increased odds of being anaemic (Table 4). Again, parity was significantly associated with anaemia in this study (Table 1). Nulliparous women were 1.92 times more likely to develop anaemia than multiparous women. This suggests that the behaviours and attitudes of primiparous and primigravid women may differ significantly from women with children or with previous pregnancies. This finding is contrary to the study by Desalegn [32] in Southwestern Ethiopia which showed that the prevalence of anaemia increased with increasing parity (nulliparity = 28%, parity 1–4 = 43.6%, and parity ≥ 5 = 53.5%; *p* < 0.01). He ascribed his findings to depleted iron stores and other nutrients during increased and repeated pregnancies and also the possibility of maternal body sharing of resources with the foetus.

According to Desalegn [32], anaemia increases with gestational age, indicating that women who wait until the third trimester to seek antenatal care are more likely to develop anaemia during pregnancy than those who seek it at an earlier gestational age. This finding is corroborated by our study in which pregnant women in the third and second trimester were 2.73 and 1.71, respectively, more likely to develop anaemia. As observed by Majoko et al. [33], women who had their first antenatal visit within the first trimester demonstrated higher compliance with recommended antenatal care. The development of anaemia is gradual and progressive, if the causative factors are not identified and treated. Therefore, pregnant women who report to the Antenatal Clinic in the first trimester are expected to have any factor that predisposes them to anaemia to be timely managed and hence the less tendency for them to be anaemic, compared to their counterparts who report beyond the first trimester.

There was a higher proportion of nonanaemic participants among those who ate single meal, compared to those who ate three daily meals. This study also showed less proportion of underweight (<22 kg/m^2^) among the anaemic participants. These findings suggest that the anaemia may not be due to malnutrition but parasitic infection or other factors.

Compared to the nonanaemic pregnant women, a higher percentage of anaemic pregnant women (70.5%) drank from well and borehole but not pipe-borne water. The major sources of water were untreated wells, boreholes, dug-out dams, and streams which harbour parasites such as* Schistosoma haematobium* and other intestinal parasites. Intestinal worm infections are common worldwide but thrive in poor communities in the tropics, where poor water supply and poor sanitation are common [34]. Drinking from wells and boreholes was over threefold more likely to expose an individual to anaemia (Table 1).

Presence of domestic animals can result in zoonotic transfer of microscopic parasites from intermediate host such as ticks to humans [35]. This study observed that keeping domestic livestock could expose one to anaemia by increasing the risk by 2.15.

## 5. Conclusion and Recommendations

This study has found that younger pregnant women had increased risk of anaemia. It also found that the risk of anaemia decreased as the number of children or pregnancies increased. Rural dwelling increased the risk of anaemia. The study found that pregnant women who reported to the Antenatal Clinic in the second trimester were more anaemic, compared to the higher percentage of the nonanaemic women, reporting in the first trimester. Therefore, the risk of anaemia increases with delay of antenatal visit. Hence it is recommended that pregnant women should seek early antenatal care since early diagnosis of anaemia is important in curbing any potential future complications that can affect maternal and foetal health.

It was also found that environmental factors such as source of water (borehole and well) and presence of domestic animals increased the risk of anaemia. Hence midwives are to educate pregnant women during antenatal sessions to depend solely on pipe-borne water. In areas without pipe-borne water, water from wells and boreholes must be treated.

Ticks on domestic animals can be prevented from transmitting zoonotic parasitic infections by spraying the skin and clothing with tick repellants such as permethrin. Hence the government, policy makers, and so forth can include tick repellants in the antenatal package of pregnant women from rural settlements to prevent haemolytic anaemia caused by parasites on domestic animals.

The limitations of our study include the small sample size and the cross-sectional nature of the study.

## Figures and Tables

**Table 1:** Sociodemographic and obstetric characteristics.

Variables	Anaemic (*n* = 200) *n* (%)	Nonanaemic (*n* = 200) *n* (%)	Crude aOR (95% CI)	*p* value
*Maternal age (mean ± SD)*	27.43 ± 6.47	27.52 ± 6.48	—	0.883

*Age group*				
<30 years	153 (76.5%)	132 (66.0%)	1.67 (1.1 to 2.6)	0.0269
≥30 years	47 (23.5%)	68 (34.0%)	Reference	

*Residency*				
Urban	80 (40.0%)	137 (68.5%)	Reference	
Rural	120 (60.0%)	63 (31.5%)	3.26 (2.162 to 4.92)	<0.0001

*Level of education*				
None	67 (33.5%)	58 (29.0%)	1.82 (1.04 to 3.19)	0.4850
Basic	100 (50.0%)	90 (45.0%)	1.75 (1.04 to 2.95)	0.3740
Higher	33 (16.5%)	52 (26.0%)	Reference	

*Occupation type*				
Unemployment	54 (27.0%)	33 (16.5%)	5.06 (2.26 to 11.33)	<0.0001
Self-employment	135 (67.5%)	133 (66.5%)	0.14 (0.06 to 1.45)	0.0012
Civil servant	11 (5.5%)	34 (17.0%)	Reference	

*Gravidity*				
Primigravida	79 (39.5%)	48 (24.0%)	2.07 (1.34 to 3.18)	0.0020
Multigravida	121 (60.5%)	152 (76.0%)	Reference	

*Parity*				
Nulliparity	82 (41.0%)	54 (27.0%)	1.879 (1.23 to 2.86)	0.0043
Multiparity	118 (59.0%)	146 (73.0%)	Reference	

*Trimester of pregnancy*				
First	69 (34.5%)	105 (52.5%)	Reference	
Second	99 (49.5%)	74 (37.0%)	2.04 (1.33 to 3.12)	0.0013
Third	32 (16.0%)	21 (10.5%)	2.32 (1.24 to 4.35)	0.0012

*Source of water*				
Pipe-borne water	59 (29.5%)	118 (59.0%)	Reference	
(well and bore hole)	141 (70.5%)	82 (41.0%)	3.44 (2.27 to 5.21)	<0.0001

*PHA*				
Yes	42 (21.0%)	29 (14.5%)	1.57 (0.93 to 2.64)	0.1159
No	158 (79.0%)	171 (85.5%)	Reference	

Values are presented as *n* (%). Comparisons between proportions in anaemic and nonanaemic groups were performed using Chi-square test. *p* < 0.05 was considered statistically significantly different. PHA: past history of anaemia.

**Table 2:** Nutrition, BMI, and presence of livestock of study participants.

Variables	Anaemic (*n* = 200) *n* (%)	Nonanaemic (*n* = 200) *n* (%)	Crude aOR (95% CI)	*p* value
*Number of meals/day*				
Three times	183 (91.5%)	172 (86%)	Reference	
Single meal	17 (8.5%)	28 (14%)	1.75 (0.92 to 3.31)	0.1128

*Source of food*				
Home only	76 (38.0%)	105 (52.5%)	Reference	
Street	5 (2.5%)	12 (6.0%)	0.57 (0.19 to 1.79)	0.4404
Both home and street	119 (59.5%)	83 (41.5%)	1.98 (1.31 to 2.97)	0.0011

*Presence of domestic livestock*				
Yes	172 (86.0%)	147 (73.5%)	2.15 (1.33 to 3.68)	0.0027
No	28 (14.0%)	53 (26.5%)	Reference	

*Body mass index*				
<22.0 kg/m^2^	99 (49.5%)	81 (40.5%)	1.44 (0.96 to 2.14)	0.0874
≥22.0 kg/m^2^	101 (50.5%)	119 (59.5%)	Reference	

Values are presented as *n* (%). Comparisons between proportions of anaemic and nonanaemic groups were performed using Chi-square test. *p* < 0.05 was considered statistically significantly different. Crude odds ratios were obtained from logistic regression analysis.

**Table 3:** Malaria parasitaemia in anaemic and nonanaemic pregnant women.

	Anaemic (*n* = 200) *n* (%)	Nonanaemic (*n* = 200) *n* (%)	*χ* ^2^, df (*p* value)
Parasitaemia			0.4611, 2 (0.794)
No Mps seen	161 (80.5%)	166 (83.0%)	
+1	35 (17.5%)	30 (15.0%)	
+2	4 (2.0%)	4 (2.0%)	
+3	—	—	

Intestinal flagellates			3.977, 2 (0.1369)
NAD	145 (72.5%)	158 (79.0%)	
+1	54 (27.0%)	39 (19.5%)	
+2	1 (0.5%)	3 (1.5%)	
+3	—	—	—

*S. mansoni *(++)	1 (0.5%)	—	—
*S. haematobium*	1 (0.5%)	—	—

Values are presented as *n* (%). Mps: malaria parasite. Comparisons between proportions in anaemic and nonanaemic groups were performed using Chi-square test. *p* < 0.05 was considered statistically significantly different.

**Table 4:** Multivariate logistic regression of factors associated with anaemia in pregnancy.

Variable	Adjusted aOR (95% CI)	*p* value
*Maternal age*		
<30 years	1.01 (CI: 1.1–2.6)	0.1101
≥30 years	Reference	

*Residency*		
Urban	Reference	
Rural	3.10 (CI: 2.16–4.91)	0.0071

*Level of education*		
None	1.88 (CI: 1.04–3.19)	0.485
Basic	1.65 (CI: 1.04–2.95)	0.712
Higher	Reference	

*Occupation status*		
Unemployment	4.76 (CI: 2.26–11.33)	<0.0001
Self-employment	0.12 (CI: 0.06–1.45)	0.0100
Civil servant	Reference	

*Gravidity*		
Primigravida	2.13 (CI: 1.34–3.18)	0.0201
Multigravida	Reference	

*Parity*		
Nulliparity	1.92 (CI: 1.23–2.86)	0.0231
Multiparity	Reference	

*Trimester of pregnancy*		
First	Reference	—
Second	1.71 (CI: 1.33–3.12)	0.0149
Third	2.73 (CI: 1.24–4.35)	0.0017

*Source of water*		
Pipe-borne water	Reference	
(well and bore hole)	2.78 (CI: 2.27–5.21)	<0.0001

*PHA*		
Yes	1.23 (CI: 0.93–2.64)	0.1378
No	Reference	

*Presence of domestic livestock*		
Yes	2.15 (CI: 1.33–3.68)	0.0019
No	Reference	

PHA: previous history of anaemia; CI: confidence interval. Logistic regression analysis was adjusted for maternal age.
